# Comparison of deep transfer learning algorithms and transferability measures for wearable sleep staging

**DOI:** 10.1186/s12938-022-01033-3

**Published:** 2022-09-12

**Authors:** Samuel H. Waters, Gari D. Clifford

**Affiliations:** 1 Department of Bioengineering, Georgia Institute of Technology, Atlanta, United States; 2 Department of Biomedical Informatics, Emory University, Atlanta, United States

**Keywords:** Transfer learning, Machine learning, Domain adaptation, Sleep staging, Wearable medical devices, EEG

## Abstract

**Background:**

Obtaining medical data using wearable sensors is a potential replacement for in-hospital monitoring, but the lack of data for such sensors poses a challenge for development. One solution is using in-hospital recordings to boost performance via transfer learning. While there are many possible transfer learning algorithms, few have been tested in the domain of EEG-based sleep staging. Furthermore, there are few ways for determining which transfer learning method will work best besides exhaustive testing. Measures of transferability do exist, but are typically used for selection of pre-trained models rather than algorithms and few have been tested on medical signals. We tested several supervised transfer learning algorithms on a sleep staging task using a single channel of EEG (AF7-Fpz) captured from an in-home commercial system.

**Results:**

Two neural networks—one bespoke and another state-of-art open-source architecture—were pre-trained on one of six source datasets comprising 11,561 subjects undergoing clinical polysomnograms (PSGs), then re-trained on a target dataset of 75 full-night recordings from 24 subjects. Several transferability measures were then tested to determine which is most effective for assessing performance on unseen target data. Performance on the target dataset was improved using transfer learning, with re-training the head layers being the most effective in the majority of cases (up to 63.9% of cases). Transferability measures generally provided significant correlations with accuracy (up to $r = -0.53$).

**Conclusion:**

Re-training the head layers provided the largest performance boost. Transferability measures are useful indicators of transfer learning effectiveness.

## Background

### At-home sleep staging

Sleep disorders such as sleep apnea and narcolepsy are diagnosed using polysomnography (PSG), a procedure where the patient spends one or more nights sleeping in a clinical setting while their EEG, ECG, heart rate, and sometimes their body temperature, blood oxygenation, and respiratory rate are recorded continuously. A clinician will then manually inspect the signals in 30-s ’epochs’ and determine whether the patient is awake, in rapid eye movement (REM) sleep, stage 1 sleep, stage 2 sleep, or stage 3 sleep according to the American Academy for Sleep Medicine rules [1]. Various statistics, such as the time spent in stage 3, time spent in REM or time spent waking up throughout the night are used as diagnostic tools. The procedure is expensive, however. A single polysomnogram can cost as much as $4000 [2] with the cost of sleep staging alone amounting to $800 [3]. Sleep stage scores can also be inconsistent, with technicians having an inter-rater reliability of 83% [4].

PSG is also unsuitable for long-term monitoring, as it typically requires special equipment and training to set up the equipment, and so patients cannot simply take recordings on themselves. Long-term monitoring of sleep disorder treatment is instead conducted using surveys of subjective sleep quality or having the patient note the time at which they went to bed or woke up each night, however, these methods are highly inaccurate compared to PSG [5] and are unable to measure certain metrics such as REM onset latency or total time spent in deep sleep. Sleep can be measured more accurately and with more detail using actigraphy [5], however this is still less accurate than PSG [5].

### Wearable medical devices

Wearable medical devices such as the Actiwatch (Philips, Amsterdam, Netherlands), Apple Watch (Apple, Cupertino, CA, USA), Sleep Profiler (Advanced Brain Monitoring, Carlsbad, CA, USA) and Dreem Headband (Dreem, Paris, France) have seen a surge in interest for their use as an alternative to in-hospital monitoring for a number of tasks, including PSG. Wearables can be used continuously in an at-home setting for lengthy periods of time by an untrained user, thereby facilitating long-term monitoring and eliminating the need for patients to be brought into a hospital.

Processing the comparatively large amount of data has driven the push for automated processing of medical data using machine learning [6–12]. However, the small amount of data available from wearable devices is an obstacle, as machine learning algorithms are immensely data-hungry. Furthermore, machine learning models trained on in-hospital recordings will not achieve good performance on wearable recordings due to a multitude of differences between in-hospital and wearable recordings, including signal quality, sensor location, available modalities, and differing pathologies between patients receiving a diagnostic polysomnogram vs those requiring long-term monitoring.

### Transfer learning

Transfer learning is the process of boosting machine learning performance on one domain (referred to as the ’target’ domain with sample set $X_t$) by pre-training the model on another, similar domain (referred to as the ’source’ domain with sample set $X_s$), thereby compensating for potentially insufficient target data by allowing the model to apply knowledge it gained from the source domain. Transfer learning poses a potential solution to the lack of data available from wearable devices. A large amount of data are available from recordings taken in a hospital using conventional medical sensors, which could potentially be used to boost machine learning performance on wearable devices.

### Limitations in current transfer learning research

There are several areas of transfer learning research which are underexplored for sleep staging. Much of supervised transfer learning is done using very simple methods such as re-training a few layers of the model (hereafter referred to as head re-training) or re-training the entire model at a smaller learning rate [13–15]. However, there are other more sophisticated transfer learning methods such as Correlation Alignment (CORAL) [16], Deep Domain Confusion (DDC) [17], and Subspace Alignment (SA) [18] which are rarely tested on sleep staging tasks. Alternatives to fully supervised domain adaptation include semi/unsupervised transfer learning [19, 20], meta-learning [21], pre-training on a related but separate task [22], and transfer learning onto individual subjects [19, 23]. Such approaches, however, have shortcomings such as not utilizing labeled data from the target domain, the need for additional labeled data that are rarely collected in clinical tasks, or the need for models to become specialized for a single subject. Therefore, the automated sleep staging field would generally benefit from a greater understanding of which fully supervised transfer learning methods would work best and when. More research comparing transfer learning techniques head-to-head is required.

Similarly, various design decisions must be made when re-training models in addition to the choice of transfer learning algorithm, such as which architecture to use, which layers to re-train and which datasets to pre-train on. There is some research on measures of transferability between datasets which are potentially useful for determining which of several pre-trained models or datasets to use in transfer learning, but again, these are rarely tested and there is little research comparing methods head-to-head. There is also little research on using transferability measures for deciding which of several transfer learning algorithms to use.

Lastly, most transfer learning research focuses on computer vision or natural language processing tasks—comparatively little research focuses on transfer learning for medical tasks, which poses a problem for medical machine learning researchers as they may erroneously use techniques which work well for computer vision or natural language processing but not on medical signals.

We tested several popular transfer learning algorithms in a supervised setting. Several publicly available in-hospital PSG datasets were used as source datasets and the target dataset was 75 recordings taken on 24 healthy adult volunteers using a wearable EEG sensor. Several transferability measures were also tested to determine which was most strongly correlated with accuracy on unseen data.

### Transfer learning algorithms

#### Head re-training

One of the simplest and more widely used transfer techniques is simply freezing every layer except for the few closest layers to the output and re-training the unfrozen layers on the target. This method was used as a baseline. After pre-training the model on the source, every layer except for a single dense layer adjacent to the output was frozen and the model was re-trained on the target.

#### CORAL

CORAL works by transforming the source data to resemble the target dataset. It works by creating a linear transformation *A* on the zero-meaned features of the source dataset which results in source features with a similar covariance matrix $C_{{\hat{s}}}$ to that of the target features $C_t$:

1 $$\begin{aligned} \begin{aligned} \min _A\left\Vert C_{{\hat{s}}} - C_t\right\Vert ^2_F = \min _A\left\Vert A^\top C_sA - C_t\right\Vert ^2_F, \end{aligned} \end{aligned}$$

where $C_s$ is the covariance matrix of the untransformed source features. It can be shown that 1 is minimized by $A^* = (U_s\Sigma _s^{+\frac{1}{2}}U_s^{\top })(U_{t[1:r]}\Sigma _{t[1:r]}^{\frac{1}{2}}U_{t[1:r]}^{\top })$, where $U_s$ is the matrix of left singular vectors of $C_s$, $U_{t[1:r]}$ is the matrix of singular vectors of the largest *r* singular values of $C_t$, $\Sigma _s^{+\frac{1}{2}}$ is the matrix square root of the Moore–Penrose pseudoinverse of the matrix of singular values of $C_s$, $\Sigma _{t[1:r]}^{\frac{1}{2}}$ is the matrix square root of the Moore–Penrose pseudoinverse of the matrix of *r* largest singular values of $C_t$ and *r* is the rank of either $C_s$ or $C_t$, whichever is smaller. Once *A* is found, it is used to transform the source data so that they more closely resemble the target data, after which training on the transformed source data proceeds as normal.

Although CORAL was originally designed to be unsupervised, it is easy to modify to be supervised by training the model on both the transformed source and untransformed target data. For this work, we also used a modified version of CORAL of our own design which takes class into account when learning the transformations. The modified CORAL will be referred to as Per-Class CORAL. Per-Class CORAL computes a different transformation $A_i$ for each class by aligning the covariances of source samples in class *i* with the covariances of target samples which are also in class *i*. Each class in the source dataset is then transformed individually.

CORAL can be applied to deep learning by performing the described transformations on learned features $\phi (x_s)$ and $\phi (x_t)$ obtained using the output from some layer of the base model, then re-training the succeeding layers of the model on the transformed features. For this work, we froze every layer of the base model from the input up to and including the convolutional layer closest to the output, then performed the CORAL algorithm on the activations from the convolutional layer closest to the output, then re-trained the unfrozen layers on the target and transformed source activations.

#### Deep domain confusion

Instead of transforming learned features, DDC works by training models in which the learned features differ little between the source on target to begin with. Training invariant features is done by adding an additional loss function equal to the maximum mean discrepancy [24] (MMD) between source and target samples within each batch.

MMD is a measure of the difference between probability distributions which finds the distance between the average kernel of the kernel embeddings $k(x_s)$ and $k(x_t)$ for source dataset $X_s$ and target dataset $X_s$:

2 $$\begin{aligned} MMD(X_s,X_t)^2 = \left\Vert \frac{1}{|X_s|}\sum _{x_s\in X_s}k(x_s) - \frac{1}{|X_t|}\sum _{x_t\in X_t}k(x_t)\right\Vert ^2_{{\mathcal {H}}_K}, \end{aligned}$$

where ${\mathcal {H}}_k$ is a reproducing kernel Hilbert space with characteristic kernel *k*. Using the property that $<x,y>_{H_k} = k(x,y)$ allows 2 to be further simplified:

3 $$\begin{aligned} MMD(X_s,X_t)^2&= \frac{1}{|X_s|^2}\sum _{i,j}^{|X_s|}\langle k(x_{s,i}),k(x_{s,j})\rangle _{H_k} \nonumber \\&\quad + \frac{1}{|X_t|^2}\sum _{i,j}^{|X_t|}\langle k(x_{t,i}),k(x_{t,j})\rangle _{H_k} \nonumber \\&\quad - \frac{2}{|X_s||X_t|}\sum _{i,j}^{|X_s|,|X_t|}\langle k(x_{s,i}),k(x_{t,j})\rangle _{H_k} \end{aligned}$$

4 $$\begin{aligned}&= \frac{1}{|X_s|^2}\sum _{i,j}^{|X_s|} k(x_{s,i},x_{s,j}) + \frac{1}{|X_t|^2}\sum _{i,j}^{|X_t|} k(x_{t,i},x_{t,j}) \nonumber \\&\quad - \frac{2}{|X_s||X_t|}\sum _{i,j}^{|X_s|,|X_t|} k(x_{s,i},x_{t,j}). \end{aligned}$$

Note that unlike other measures of distance between probability distributions such as KL-divergence or Wasserstein distance, MMD can be computed directly from samples, and does not require an estimate of the probability density. Time consuming and potentially inaccurate computations of probability density such as through kernel density estimation are thus unnecessary. 4 is computed with quadratic time complexity, but if the samples $x_s$ and $x_t$ are independent and identically distributed and $|X_s| = |X_t| = n$, an unbiased estimate can be used which can be computed in linear time [24]:

5 $$\begin{aligned} \begin{aligned} MMD(X_s,X_t)^2&= \frac{2}{n}\sum _{i}^{n/2} k(x_{s,2i-1},x_{s,2i}) + \frac{2}{n}\sum _{i}^{n/2} k(x_{t,2i-1},x_{t,2i}) \\&\quad - \frac{2}{n}\sum _{i}^{n/2} k(x_{s,2i-1},x_{t,2i}) - \frac{2}{n}\sum _{i}^{n/2} k(x_{t,2i-1},x_{s,2i}). \end{aligned} \end{aligned}$$

The efficiency at which MMD can be calculated makes it useful in machine learning algorithms in which it may need to be computed repeatedly.

In DDC, the MMD of activations at one or several layers is calculated between source and target samples in each batch and used as a loss function in addition to the standard cross-entropy loss. In doing so, the neural network is incentivized to learn features which are very similar between source and target, and so the model can be simultaneously trained on both source and target datasets without losing accuracy on the target. To maintain consistency with the other transfer learning methods we implemented, we chose the final convolutional layer as the layer at which to calculate the MMD loss.

#### Subspace alignment

SA works by projecting both the source and target data onto two lower-dimensional linear subspaces, then using a linear transformation *M* to align the source subspace with the target subspace. The lower-dimensional subspaces for both the source and target are found through principal component analysis (PCA) using a fixed dimensionality *d*. *M* can be efficiently computed using:

6 $$\begin{aligned} M = V_s^TV_t, \end{aligned}$$

where $V_s$ and $V_t$ are matrices of the basis vectors for the source and target subspaces. A new source sample $x_s$ can then transformed into the target subspace using $x_s^TV_sM$. In the fully or semi-supervised setting, the source and target samples are transformed into the target subspace, after which a model can be trained on both. As with CORAL, we froze each layer in the base model from the input to and including the convolutional layer closest to the output, then performed SA on the activations from the convolutional layer closest to the output, then re-trained the unfrozen layers on the transformed activations. We choose the dimensionality of the transformed features to be 100, which we determined via grid search on a subset of 7 target subjects.

### Transferability measures

#### Log expected empirical prediction

Log expected empirical prediction (LEEP) is a measure of transferability which creates a simple Bayesian classifier which attempts to classify target samples based on the outputs of a model trained on the source dataset:

7 $$\begin{aligned} {\hat{P}}(y|z)&= \frac{{\hat{P}}(y,z)}{{\hat{P}}(z)} \end{aligned}$$

8 $$\begin{aligned}&= \frac{\frac{1}{n}\sum _{i:y_i=y}\theta (x_{t,i})_z}{\frac{1}{n}\sum ^n_{i}\theta (x_{t,i})_z}, \end{aligned}$$

where *z* are the label outputs of the pre-trained model, $\theta (x_{t,i})_z$ is the model’s estimated probability that sample $x_{t,i}$ has label *z*, and $\sum _{i:y_i=y}$ indicates all the samples whose true label is *y*. LEEP $T(\theta )$ for a model $\theta$ is equal to the average log-likelihood of the Bayesian classifier on the target domain:

9 $$\begin{aligned} T(\theta ) = \frac{1}{n}\sum ^n_i\log \left( \sum _z {\hat{P}}(y_i|z)\theta (x_{t,i})_z\right) . \end{aligned}$$

LEEP can be considered a measure of how well the Bayesian classifier performs on the target dataset, which by extension indicates how well the model will perform on the target dataset after a small amount of re-training, since the Bayesian model performs classification using the pre-trained model’s outputs.

#### H-score

Bao *et al.* [25] show that the following is minimized when a given feature extractor $\phi$ is optimal:

10 $$\begin{aligned} H(\phi ) = tr(cov(\phi (X_t))^{-1}cov({\mathbb {E}}_{P(X_t|Y)}[\phi (X_t)|Y])). \end{aligned}$$

$H(\phi )$ can thus be used as indicator of how well suited a classifier with learned feature extractor $\phi$ is for a particular dataset. Note that the exact value of $H(\phi )$ will depend on the dataset, and thus that a higher H-score on one target dataset does not mean a model re-trained on that dataset will necessarily perform better than a model trained on another dataset with a lower H-score. Bao *et al.* [25] also derives a value they dub *transferability* which can be compared across different target datasets by normalizing H-score by its theoretical minimum possible value, however it is not necessary to do so to determine which of several possible models will perform best on a fixed dataset, and the calculation of transferability requires a more time-consuming iterative procedure. We therefore performed all testing using H-score and not transferability in order to reduce computation time.

#### Hypothesis margin

One practical advantage of LEEP and H-score is that they only require the model and the target dataset to compute—it is not necessary to have any data from the source dataset. However, this is also a disadvantage from a theoretical standpoint as it is more difficult to interpret what characteristics of the source dataset make it effective for pre-training. We thus propose using several measures of statistical characteristics of datasets which have more concrete interpretations.

Hypothesis margin is a measure of the margins between sets of points with differing labels, which has been used in feature selection [26–29] and in loss functions for machine learning [30]. The hypothesis margin *M*(*x*) for a single point *x* is:

11 $$\begin{aligned} M(x) = \frac{1}{2}(\left\Vert x - nearmiss(x)\right\Vert - \left\Vert x - nearhit(x)\right\Vert ), \end{aligned}$$

where *nearmiss*(*x*) is the nearest point to *x* which is in a different class from *x* and *nearhit*(*x*) is the nearest point to *x* which is in the same class. We used the average hypothesis margin $\bar{M}(X_t,X_s)$ to study the margin between learned features extracted from points in the source dataset and learned features extracted from points in the target dataset using the feature extractor $\phi$ from a model pre-trained on the source dataset:

12 $$\begin{aligned}&\bar{M}(X_t,X_s) = \frac{1}{2(|X_t| + |X_s|)}\sum _{x\in X_t\cup X_s} \left\Vert \phi (x) - \phi (nearmiss(x))\right\Vert \end{aligned}$$

13 $$\begin{aligned}&- \left\Vert \phi (x) - \phi (nearhit(x))\right\Vert . \end{aligned}$$

In this work, we downsample $X_t$ and $X_s$ by a factor of 10 to reduce the computational resources used in computing the distances between each pair of samples. We hypothesized that when there is less of a margin between the learned features from the source and target dataset, performance on the target dataset will be better because it requires less adjustment for the model to achieve good performance.

#### Silhouette score

Similarly, we hypothesized that when there is a greater degree of overlap between learned features of the source and target dataset, transfer learning performance will be better due to the smaller amount of adjustment necessary to make to the model. We measured degree of overlap using silhouette score:

14 $$\begin{aligned} S(X) = \frac{1}{n}\sum ^n_{x \in X}\frac{A(x) - B(x)}{\max \{A(x),B(x)\}}, \end{aligned}$$

where *A*(*x*) is the average L2 distance from point *x* to every other point in the same class and *B*(*x*) is the average L2 distance from *x* to every point in a different class.

Silhouette score is a measure of the degree of overlap between sets of points of differing classes or clusters, and is most often used in evaluating the quality of clustering algorithms [31, 32].

Silhouette score between the learned features from the source and target $S(\phi (X_t),\phi (X_s))$ is:

15 $$\begin{aligned} S(\phi (X_t),\phi (X_s)) = \frac{1}{|X_t| + |X_s|}\sum _{x \in X_t\cup X_s}\frac{A(\phi (x)) - B(\phi (x))}{\max \{A(\phi (x)),B(\phi (x))\}}. \end{aligned}$$

In this case, $A(\phi (x))$ is then the average L2 distance from $\phi (x)$ to every point in the same dataset and $B(\phi (x))$ is the average L2 distance from $\phi (x)$ to every point in the other dataset.

#### Target density around source

Target density around source [18] (TDAS) is a measure of the local density of target samples within some neighborhood of the source samples, and is mainly intended for use in nearest-neighbor models [18]. Let $sim(x_s,x_t) = (x_sV_sM)(x_tV_t)^T$ for $x_s$ and $x_t$ are a source and target sample, $V_s$, $V_t$ are the subspace bases found through PCA on the source and target, and *M* is a transformation for aligning the source and target basis vectors as explained in Section 1.5.4. $sim(x_s,x_t)$ can be considered a measure of similarity between a source and target sample following alignment of their lower-dimensional projections. To measure the transferability between two datasets, TDAS is defined as the average number of target samples that have similarity of at least $\epsilon$ to a given target sample:

16 $$\begin{aligned} TDAS = \frac{1}{|X_s|}\sum _{x_s\in X_s}|{x_t\in X_t|sim(x_s,x_t)\ge \epsilon }|. \end{aligned}$$

We chose $\epsilon$ to be the median Euclidean distance between samples in the target dataset *m* multiplied by either .1, 1 or 10. The reason why we use $\epsilon$ at 3 different values is to evaluate the sensitivity of TDAS to $\epsilon$. The reason we chose to tie $\epsilon$ to the median value instead of using some fixed value was so that the effectiveness of TDAS would be more stable across target datasets of differing pairwise distances between samples.

#### Maximum mean discrepancy

As explained in Section 1.5.3, MMD is a measure of the similarity between probability distributions which is used in machine learning, both for training algorithms [17, 33] and in making design decisions for transfer learning [17]. We use the radial basis function for the kernel with width parameter $\gamma$ parameter equal to .1$\Gamma$, 1$\Gamma$ or 10$\Gamma$, where $\Gamma = -1/(2*M)$. Using the value $\gamma = 1\Gamma$ is the median heuristic [24, 34] for calculating $\gamma$, but as with TDAS we computed MMD using the parameter value increased or decreased in order to observe the sensitivity of the MMD measure to $\gamma$.

## Results

**Fig. 1:**
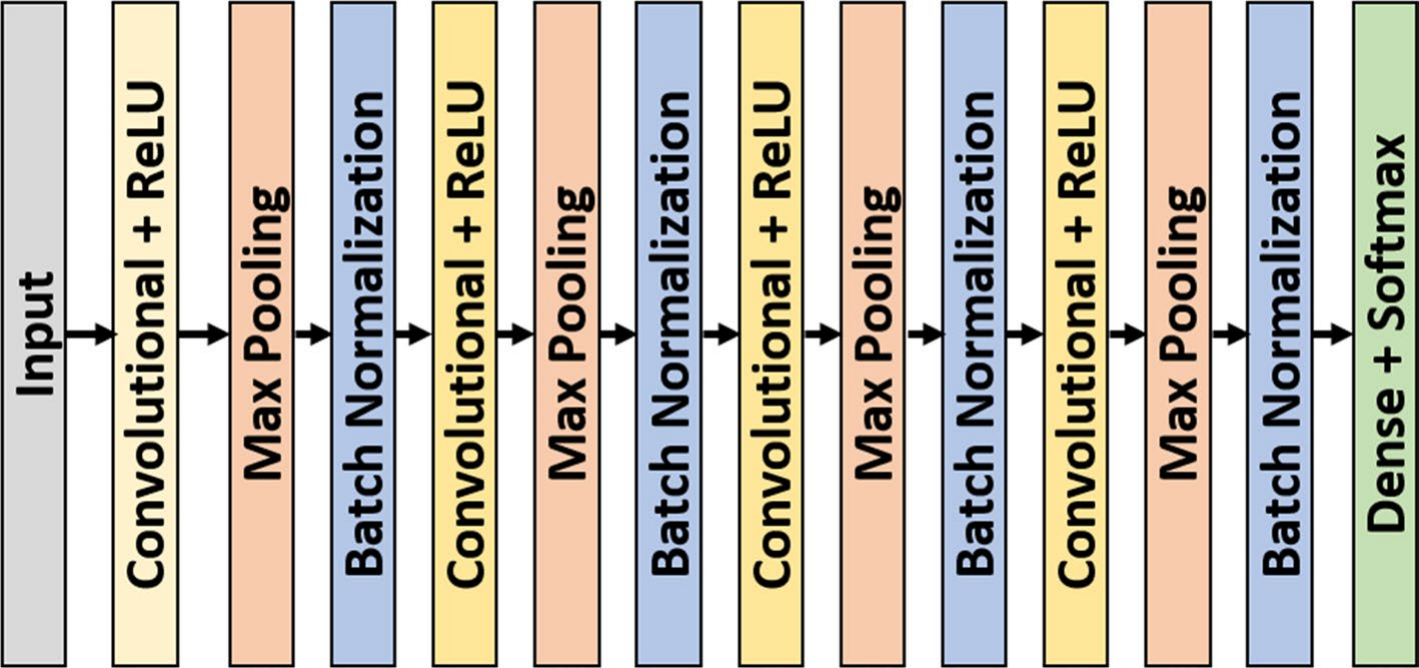
Architecture of base model

**Fig. 2:**
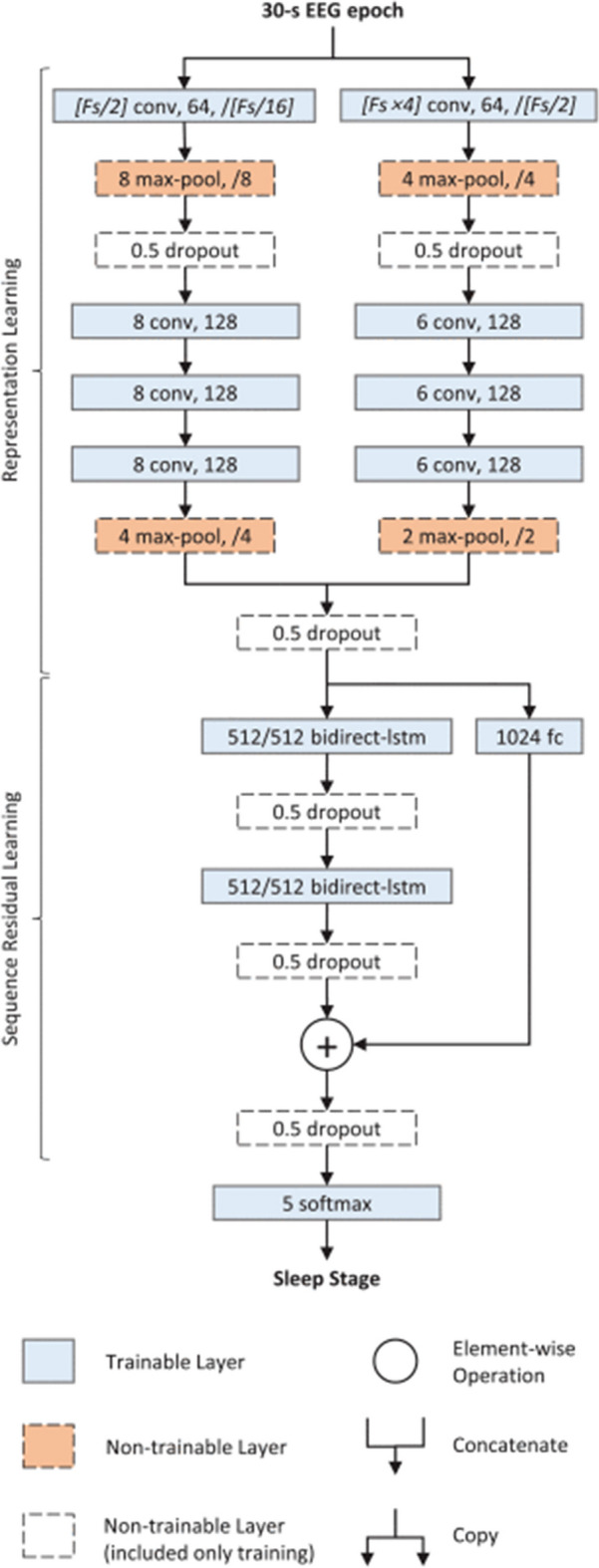
Architecture of open-source model, DeepSleepNet [43]. Image courtesy of Supratak *et al*. [43]

Several publicly available PSG datasets using standard 10–20 scalp montages were used as source domains: Sleep Heart Health Study dataset (SHHS) [35, 36], the Computing in Cardiology Challenge 2018 dataset (CiCC) [37, 38], the Institute of Systems and Robotics, University of Coimbra dataset (ISRUC) [39], the Osteoporotic Fractures in Men Study dataset (MrOS) [35, 40], The Montreal Archive of Sleep Studies (MASS) [41], and the Wisconsin Sleep Cohort (WSC) [35, 42]. The target dataset consisted of 75 recordings from 24 subjects we obtained using an X4 Sleep Profiler (Advanced Brain Monitoring, Carlsbad, CA)—a commercially available wearable EEG sensor. Two architectures were used: a novel and relatively simple bespoke (13-layer convolutional neural network (CNN)) model that we designed for use on resource-constrained body-worn systems (Fig 1); and a more computationally intensive contemporary open-source algorithm called DeepSleepNet [43] (Fig 2). This algorithm was selected due to its state-of-the-art performance on sleep staging tasks, its open architecture, and for its frequent use as a basis of comparison by other researchers [7, 22, 44–47]. DeepSleepNet contains 35 layers, including both convolutional and long short-term memory (LSTM) layers [43].

### Performance on bespoke CNN

**Table 1:** Cross-validation % accuracy (average ± standard deviation) obtained using each algorithm and source dataset to re-train the CNN

	CiCC	ISRUC	MASS	SHHS	WSC	MrOS
Head Re-train	78.1 ± 6.3	75.9 ± 6.8	75.5 ± 6.9	76.0 ± 6.5	76.5 ± 6.4	76.2 ± 8.9
Subspace alignment	53.7 ± 14.1	48.0 ± 12.6	43.3 ± 11.8	45.9 ± 11.3	45.0 ± 12.0	36.7 ± 12.9
CORAL	78.0 ± 6.3	76.1 ± 6.3	76.8 ± 6.1	75.5 ± 6.3	76.6 ± 6.0	77.1 ± 6.4
Per-Class CORAL	78.0 ± 5.7	75.7 ± 6.8	76.4 ± 5.9	75.6 ± 6.2	76.0 ± 6.1	77.2 ± 5.9
DDC	75.7 ± 8.3	77.7 ± 6.7	77.4 ± 8.5	78.8 ± 7.5	75.9 ± 9.1	79.0 ± 6.3

**Table 2:** Cross-validation Cohen’s $\kappa$ (average ± standard deviation) obtained using each algorithm and source dataset to re-train CNN

	CiCC	ISRUC	MASS	SHHS	WSC	MrOS
Head Re-train	0.689 ± 0.086	0.659 ± 0.093	0.652 ± 0.092	0.661 ± 0.087	0.669 ± 0.085	0.667 ± 0.115
Subspace alignment	0.332 ± 0.183	0.279 ± 0.151	0.146 ± 0.139	0.230 ± 0.140	0.183 ± 0.137	0.153 ± 0.136
Per-Class CORAL	0.690 ± 0.077	0.660 ± 0.090	0.666 ± 0.079	0.658 ± 0.083	0.663 ± 0.081	0.681 ± 0.080
CORAL	0.689 ± 0.085	0.663 ± 0.084	0.672 ± 0.081	0.655 ± 0.084	0.669 ± 0.080	0.679 ± 0.086
DDC	0.660 ± 0.109	0.686 ± 0.088	0.682 ± 0.111	0.703 ± 0.098	0.663 ± 0.118	0.704 ± 0.085

**Table 3:** Accuracy, $\kappa$, and % of cases where each algorithm outperformed all other algorithms for bespoke CNN

Algorithm	Average ± standard deviation % accuracy	Average ± standard deviation Cohen’s $\kappa$	% of cases where algorithm was best
Head Re-train	76.4 ± 7.1	0.666 ± 0.095	22.2
Subspace alignment	45.4 ± 13.5	0.220 ± 0.163	0.7
Per-Class CORAL	76.5 ± 6.2	0.670 ± 0.083	9.0
CORAL	76.7 ± 6.2	0.671 ± 0.084	14.6
**DDC**	**77.4** ± **7.9**	**0.683** ± **0.104**	**52.1**

Table 1 lists the leave-one-subject-out cross-validation accuracies attained on the target dataset when using each source dataset for pre-training and each transfer learning algorithm for re-training. Table 2 lists the leave-one-subject-out Cohen’s $\kappa$ values. Table 3 lists the fraction of instances in which a particular learning algorithm outperformed other techniques. Every transfer learning algorithm had better than or equivalent performance to the baseline method of head re-training except for SA, which did noticeably worse. CORAL and Per-Class CORAL were not significantly better than head re-training ($p > 0.05$, $n = 144$). DDC (indicated in bold) was significantly better than head re-training ($p < 0.05$, $n = 144$). DDC also performed better than any other method a majority (52.1%) of the time. As an additional baseline, we also trained the model from random initialization on the target dataset without pre-training and achieved an accuracy of 77.0%, which is slightly better than head re-training but slightly below the highest achieving method of DDC. We also tested re-training the entire model instead of just the head, which achieved a similar performance of 77.3%.

### Correlating transfer learning performance with transferability measures

**Table 4:** Correlations of each transferability measure with CNN accuracy for individual algorithms as well as overall

Measure	Head Re-train, $n = 144$	CORAL, $n = 144$	Per-Class CORAL, $n = 144$	SA, $n = 144$	DDC, $n = 144$	Overall, $n = 720$
LEEP	− 0.03	0.24**	0.28***	0.30***	− 0.11	− 0.07
H-score	0.36***	0.47***	0.47***	− 0.05	− 0.10	0.23***
Hypothesis margin	− 0.07	0.05	0.07	− 0.11	0.15	0.08
Silhouette score	− 0.31	− 0.21*	− 0.21*	0.13	0.07	− 0.11*
MMD, $\gamma = 0.1\Gamma$	− 0.25**	− 0.14	− 0.11	− 0.36***	0.33***	− 0.11**
MMD, $\gamma = 1\Gamma$	0.07	0.08	0.03	− 0.44***	0.05	− 0.04
MMD, $\gamma = 10\Gamma$	0.16	0.29***	0.25**	− 0.53***	0.07	0.05
TDAS, $\epsilon = 0.1m$	0.17*	0.24**	0.28***	− 0.17*	0.01	0.11***
TDAS, $\epsilon = 1m$	− 0.36***	− 0.42***	− 0.42***	0.19***	0.08	− 0.18***
TDAS, $\epsilon = 10m$	− 0.35***	− 0.41***	− 0.42*	0.19*	0.07	− 0.18***

*p < 0.05

**p < 0.01

***p < 0.001

Table 4 lists the Spearman’s correlations of each transferability measure with the accuracy of a bespoke CNN model re-trained using each transfer learning method. Every transferability measure except hypothesis margin achieved significant ($p < 0.05$) correlations with accuracy when using at least one transfer learning algorithm. This continued to be true even after a Bonferroni adjustment ($p < 0.0017$). H-score achieved the highest overall correlation ($r = 0.23$). Measures were generally more strongly correlated with accuracies within individual algorithms than across all algorithms. The correlation strengths of MMD and TDAS also varied by the value of their respective parameters.

### Effect of re-training layers on performance

To test the sensitivity of each algorithm to the layer on which the domain transformation is applied, we re-tested the bespoke CNN when re-training additional layers (Tables 5 and 6). See Section 5.5 for training details.

**Table 5:** % Accuracy (average ± standard deviation) obtained for each algorithm and source when re-training additional layers of CNN

	CiCC	ISRUC	MASS	SHHS	WSC	MrOS
Head Re-train	79.0 ± 7.3	78.2 ± 7.2	79.0 ± 7.3	78.3 ± 7.3	79.1 ± 7.3	78.9 ± 7.4
Subspace alignment	56.1 ± 6.7	56.3 ± 6.8	54.2 ± 6.8	57.5 ± 6.8	52.5 ± 6.8	48.4 ± 6.9
CORAL	80.0 ± 8.4	78.4 ± 8.4	79.0 ± 8.4	78.2 ± 8.5	78.9 ± 8.5	78.7 ± 8.7
Per-Class CORAL	78.2 ± 7.3	75.8 ± 7.7	78.0 ± 7.7	77.6 ± 7.8	77.5 ± 7.8	77.3 ± 7.8
DDC	64.3 ± 9.9	65.3 ± 9.8	59.4 ± 9.8	64.2 ± 9.9	63.6 ± 9.8	62.6 ± 10.0

**Table 6:** Cohen’s $\kappa$ (average ± standard deviation) obtained for each algorithm and source when re-training additional layers of CNN

	CiCC	ISRUC	MASS	SHHS	WSC	MrOS
Head Re-train	0.704 ± 0.099	0.692 ± 0.098	0.703 ± 0.098	0.694 ± 0.099	0.704 ± 0.098	0.702 ± 0.100
Subspace alignment	0.364 ± 0.003	0.389 ± 0.003	0.326 ± 0.003	0.404 ± 0.003	0.298 ± 0.004	0.305 ± 0.004
Per-Class CORAL	0.691 ± 0.058	0.657 ± 0.118	0.691 ± 0.142	0.684 ± 0.162	0.681 ± 0.180	0.681 ± 0.200
CORAL	0.718 ± 0.062	0.696 ± 0.222	0.704 ± 0.303	0.693 ± 0.362	0.702 ± 0.402	0.700 ± 0.438
DDC	0.522 ± 0.000	0.535 ± 0.001	0.469 ± 0.002	0.525 ± 0.002	0.516 ± 0.002	0.498 ± 0.003

**Table 7:** Accuracy, $\kappa$, and % of cases where each algorithm outperformed others when re-training additional layers of CNN

Algorithm	Average ± standard deviation % accuracy	Average ± standard deviation Cohen’s $\kappa$	% of cases where algorithm was best
**Head Re-train**	**78.7** ± **6.4**	**0.700** ± 0**.087**	**39**.**6**
Subspace alignment	54.2 ± 17.0	0.348 ± 0.216	2.1
Per-Class CORAL	77.4 ± 6.9	0.681 ± 0.096	12.5
CORAL	78.9 ± 6.4	0.702 ± 0.086	31.3
DDC	63.2 ± 20.5	0.511 ± 0.24	13.2

As with Section 2.0.1, Table 7 lists the fraction of instances in which a particular learning algorithm outperformed other techniques. All algorithms performed better except DDC, which performed worse. With the exception of DDC, the algorithms’ performances relative to each other are similar to when re-training a smaller number of layers (i.e., Head Re-train, CORAL, and Per-Class CORAL performed similarly while SA performed the worst). Head Re-train outperformed all other transfer learning algorithms on the largest number of cases, but did not have a significantly higher average accuracy than CORAL ($p > 0.05$). Head Re-train, CORAL, and Per-Class CORAL also now outperformed the baseline of training from random initialization without transfer learning. Table 8 lists the correlations between each measure of transferability and accuracy achieved when re-training a larger number of layers. Correlations are generally lower, but TDAS and H-score continue to be the most strongly correlated with accuracy and achieve statistical significance in half of all cases, even after applying Bonferroni correction ($p < 0.001$).

**Table 8:** Correlations of each transferability measure with CNN accuracy when re-training additional layers of CNN

Measure	Head Re-train, $n = 144$	CORAL, $n = 144$	Per-Class CORAL, $n = 144$	SA, $n = 144$	DDC, $n = 144$	Overall, $n = 720$
LEEP	− 0.14	− 0.19*	− 0.12	0.08	0.03	− 0.07
H-score	0.17*	0.35***	0.31***	− 0.03	− 0.07	0.14***
Hypothesis margin	0.05	0.01	0.10	− 0.15	− 0.05	− 0.01
Silhouette score	− 0.05	− 0.19*	− 0.09	− 0.09	0.00	− 0.08
MMD, $\gamma = 0.1\Gamma$	0.14	0.04	− 0.03	− 0.19*	− 0.01	− 0.01
MMD, $\gamma = 1\Gamma$	0.14	0.12	0.03	− 0.14	− 0.02	0.03
MMD, $\gamma = 10\Gamma$	0.06	0.08	0.01	− 0.16	0.04	0.00
TDAS, $\epsilon = 0.1m$	− 0.07	− 0.05	− 0.02	− 0.05	0.01	− 0.03
TDAS, $\epsilon = 1m$	0.24**	0.42***	0.28***	0.15	0.06	0.15***
TDAS, $\epsilon = 10m$	− 0.10	0.24**	− 0.09	0.10	0.01	− 0.06

*p < 0.05

**p < 0.01

***p < 0.001

### Testing on differing network architectures

We also tested each transfer learning algorithm and transferability measure on the open-source sleep staging model DeepSleepNet [43] to determine how performance is affected by changes in the network architecture (Tables 9 and 10). Note that DDC was not tested on DeepSleepNet because the derivation of DDC assumes that each sample is presented in a random order during training [17, 24], an assumption which is violated in recurrent models.

**Table 9:** % Accuracy (average ± standard deviation) obtained for each algorithm and source using DeepSleepNet

	CiCC	ISRUC	MASS	SHHS	WSC	MrOS
Head Re-train	72.4 ± 9.6	59.9 ± 9.0	63.2 ± 9.4	62.3 ± 8.9	59.9 ± 8.4	64.2 ± 10.9
Subspace alignment	62.0 ± 6.8	55.3 ± 9.6	53.1 ± 11.4	52.4 ± 8.2	50.8 ± 9.2	53.5 ± 11.4
CORAL	70.6 ± 9.3	60.5 ± 8.8	61.8 ± 9.6	60.3 ± 8.1	58.8 ± 7.4	62.9 ± 8.8
Per-Class CORAL	70.8 ± 8.5	57.0 ± 8.8	62.0 ± 8.8	59.8 ± 7.4	57.7 ± 9.2	60.6 ± 10.8
DDC	N/A	N/A	N/A	N/A	N/A	N/A

**Table 10:** Cohen’s $\kappa$ (average ± standard deviation) obtained for each algorithm and source using DeepSleepNet

	CiCC	ISRUC	MASS	SHHS	WSC	MrOS
Head Re-train	0.611 ± 0.127	0.432 ± 0.133	0.477 ± 0.133	0.466 ± 0.123	0.432 ± 0.121	0.493 ± 0.140
Subspace alignment	0.446 ± 0.131	0.345 ± 0.135	0.301 ± 0.155	0.293 ± 0.113	0.264 ± 0.119	0.342 ± 0.150
Per-Class CORAL	0.584 ± 0.117	0.393 ± 0.118	0.449 ± 0.127	0.424 ± 0.106	0.391 ± 0.129	0.450 ± 0.137
CORAL	0.586 ± 0.119	0.437 ± 0.127	0.452 ± 0.134	0.431 ± 0.113	0.412 ± 0.107	0.478 ± 0.112
DDC	N/A	N/A	N/A	N/A	N/A	N/A

**Table 11:** Accuracy, $\kappa$, and % of cases where each algorithm outperformed others when using DeepSleepNet

Algorithm	Average ± standard deviation % accuracy	Average ± standard deviation Cohen’s $\kappa$	% of cases where algorithm was best
Head Re-train	63.7 ± 4.2	0.637 ± 0.144	63.9
Subspace alignment	54.5 ± 3.6	0.545 ± 0.147	1.4
Per-Class CORAL	61.3 ± 4.6	0.613 ± 0.139	13.2
CORAL	62.5 ± 3.9	0.625 ± 0.132	19.4
DDC	N/A	N/A	N/A

As with the bespoke CNN, SA exhibited the lowest performance and CORAL, Per-Class CORAL and Head Re-train all exhibited similar (higher) performance (Table 11), but with Head Re-train now being statistically significantly better than CORAL ($p < 0.01$). However, no transfer learning method was able to out-perform the baseline of training from random initialization without transfer learning, which achieved an accuracy of 74.5%.

**Table 12:** Correlations of each transferability measure with DeepSleepNet accuracy for individual algorithms as well as overall

Measure	Head Re-train, $${n = 144}$$	CORAL, $$ {n = 144}$$	Per-Class CORAL, $${n = 144}$$	SA, $$ {n = 144}$$	DDC, $${n = 144}$$	Overall, $${n = 576}$$
LEEP	0.01	0.13	0.11	0.36***	N/A	0.15****
H-score	0.12	0.15	0.23*	0.26***	N/A	0.19***
Hypothesis margin	− 0.32***	− 0.16*	− 0.27	0.10	N/A	− 0.16***
Silhouette score	− 0.31***	− 0.21*	− 0.21*	0.13	0.07	− 0.11*
MMD, $\gamma = 0.1\Gamma$	− 0.40***	− 0.31***	− 0.46***	− 0.18*	N/A	− 0.34***
MMD, $\gamma = 1\Gamma$	− 0.28***	− 0.29***	− 0.40***	− 0.29***	N/A	− 0.31***
MMD, $\gamma = 10\Gamma$	− 0.12	-0.13	− 0.28***	- 0.13	N/A	− 0.17***
TDAS, $\epsilon = 0.1m$	0.48***	0.47***	0.40***	0.30***	N/A	0.40***
TDAS, $\epsilon = 1m$	0.38***	0.37***	0.31***	0.25**	N/A	0.33***
TDAS, $\epsilon = 10m$	− 0.11	− 0.09	− 0.10	0.07	N/A	− 0.06

*p < 0.05

**p < 0.01

***p < 0.001

Correlations between accuracy and each transferability measure were highest for TDAS and MMD (Table 12). Unlike with the bespoke model, H-score’s correlation with accuracy no longer achieves Bonferroni-adjusted significance for any algorithms except for SA, but is still significant in the overall case.

## Discussion

Our findings are twofold: (1) we evaluated the performance of several transfer learning algorithms head-to-head on a sleep staging task and (2) we evaluated how well several measures of transferability work for assessing the accuracy achievable when using a particular source dataset and transfer learning algorithm.

Transfer learning techniques were tested and compared on a sleep staging task in which a model pre-trained on clinical data was re-trained on data from a wearable device using one of two possible models, two possible sets of layers to re-train, and five different algorithms. Out of all source datasets, architectures and algorithms tested, the highest accuracy and Cohen’s $\kappa$ achieved was 80.0% and .718, respectively, which were obtained using CORAL on the bespoke model when re-training more (four) layers.

With the exception of DDC, the relative performance of each transfer learning algorithm was consistent across different conditions. CORAL and Per-Class CORAL both performed similarly to the baseline performance. SA was the poorest performing in all cases. We speculate that the reduced effectiveness of SA occurs because SA involves projecting the learned features onto a lower-dimensional linear subspace which risks the loss of critical information. DDC usually obtained the highest accuracy when applied to a layer close to the output, but not when applied to a layer further from the output, suggesting that DDC can be effective but is highly sensitive to the layer to which it is applied, and so tuning may be necessary. The higher performance when the loss is applied to layers closer to the output makes sense given the principle behind DDC. That is, DDC works by incentivizing the model to learn a similar hidden-layer representation across both source and target datasets, so if the layer it is applied to already generalizes well between datasets (and layers closer to the input have indeed been found to learn simpler, more generalizable features [48–50]), it may be of limited benefit. In contrast, head re-training resulted in the best or second best performance in all training conditions, suggesting it is the most robust choice for obtaining a good (even if not necessarily the best) performance when time and resources available for hyperparameter tuning are limited.

Unlike with the bespoke CNN, All transfer learning methods reduced performance when using DeepSleepNet. This could be attributable to the greater depth of DeepSleepNet relative to the bespoke CNN, so re-training and domain adaptation at the output layer was inadequate for compensating for how much information from the source dataset had been encoded into the network.

The correlations between accuracy and several measures of transferability were also assessed to determine whether these measures were potentially useful for transfer learning design. Most measures attained significant correlations on at least some transfer learning algorithms, which is consistent with previous research [14, 17, 18, 25]. The search for effective measures of transferability is relevant to machine learning engineers seeking to reduce development time. Using transferability measures to guess which transfer learning methods will work best could avoid the need for exhaustive testing.

When performing transfer learning on the bespoke model with a smaller number of re-trainable layers, the transferability measure most correlated with accuracy overall was the H-score, and the second most correlated was TDAS. On the other two testing conditions however (i.e., transfer learning on DeepSleepNet and transfer learning on the bespoke model with a larger number of re-trainable layers), TDAS outperformed H-score, especially when using DeepSleepNet. Therefore, although the H-score may perform well in some cases, its performance is inconsistent and so TDAS may be the more robust option. TDAS did exhibit some sensitivity to choice of parameter $\epsilon$, however Fernando *et al.’s*. recommendation of setting $\epsilon$ to the median Euclidean distance between target samples [18] outperformed other choices for $\epsilon$ in most cases and was the second best choice in the remaining cases, and so can be considered a good heuristic. Despite the high overall correlations of TDAS and H-score, other measures still achieved higher correlations with accuracy for specific algorithms, and so it may be more advisable to make design decisions (such as on which source dataset to pre-train) using the transferability measure most suitable for a particular learning algorithm.

It is important to note that no single transfer learning method performs best in all cases. DDC, for example showed the strongest performance overall when re-training with a smaller number of layers, but still performed worse than the baseline when re-training with a larger number of layers.

Similarly, no transferability measure had significant correlations with the performance of all transfer learning algorithms. H-score, for example was significantly correlated with performance on every transfer learning algorithm on the bespoke architecture when re-training a smaller number of layers except DDC. The poor correlation with DDC is likely because H-score assumes a fixed feature extractor [25], whereas DDC involves fine-tuning the feature extraction layers. We can thus conclude that H-score would be effective as an indicator when using CORAL, but one should use some other transferability measure such as MMD or TDAS when using DDC. Furthermore, H-score achieved significant correlations for very few algorithms when applied to DeepSleepNet, which again, may result from the state-dependence of the LSTM layers violating H-score’s assumptions.

Since no transfer learning algorithm or transferability measure performed best in all cases, the results here cannot be taken as a replacement for exhaustive testing. The only guaranteed way to determine which of several methods will work best is to test them. However, when there is limited time and resources available, the results presented in this work can be used to narrow down the list of possible methods to a few with a higher probability of success. When there is insufficient time to experiment with multiple possible transfer learning algorithms, our results indicate that head re-training can lead to the most robust results (i.e., it consistently performs comparably to, or better than other approaches). If multiple pre-trained models are available, our results favor choosing the model which attains the highest TDAS score on the target dataset. When there is limited time for testing, but there are a large number of possible transfer learning algorithms and/or pre-trained models to choose from, we recommend calculating the TDAS value of all possible combinations of algorithms and pre-trained models, selecting the highest-scoring combinations, and directly testing them to determine which achieves the highest performance.

More research is needed to determine why some algorithms work better in some cases but not others, but we speculate that performance varies according to whether the fundamental assumptions of the algorithms are met. CORAL works well when the target distribution can be approximated by a linear transformation of the source distribution. DDC works well when the domain shift between the source and target is amplified along the layers of the neural network, possibly due to overfitting to the source dataset at layers closer to the output, in which case the model benefits from an additional loss function which punishes differences in the activations. Subspace Alignment works well when the relevant features lie on a lower-dimensional manifold, in which case projection onto this manifold does not cause significant loss of information.

When the assumptions of the more sophisticated transfer learning methods are not met, the additional constraints, operations and loss functions employed by such algorithms can instead cause loss of information or steer the model away from an optimal solution, in which case the simplest method of head re-training provides the highest performance.

The same is also true for the transferability measures. Performance depends on whether the assumptions of the measures are met. The H-score works well when the network layers closer to the input are fixed, but this assumption is violated by transfer learning methods which involve re-training such layers (such as DDC) or when those layers have state-dependence (such as when using an LSTM layer).

The randomization of samples in the MMD approach may reduce its reliability as a transferability measure through the introduction of stochasticity, but MMD can still out-perform deterministic measures such as TDAS in some cases despite TDAS working better in general. TDAS tallies the number of target samples in close proximity to source samples, but target samples far away from any source samples have little effect on the TDAS score. MMD on the other hand takes all samples into account. As a result, MMD may work better as a measure of transferability when there are many target samples which are distant from source samples or the data exhibit extreme outliers, even if TDAS works better in general. MMD may also out-perform TDAS when training via DDC, as DDC explicitly minimizes MMD between source and target, and so a large MMD may indicate that performance improvements can be made by an algorithm designed to reduce the value of MMD.

This is the first work we know of to test transferability measures across multiple transfer learning algorithms, and is also the first work we know of to evaluate transferability measures on a sleep staging task. Our findings also add to the body of research on the use of automation for wearable medical sensors, particularly regarding the use of transfer learning to boost performance [7, 51–53]. In particular, our work adds to the body of work on fully supervised transfer learning without the need for tuning models to specific patients.

One limitation was that the test subjects were healthy adults of similar age, and so more testing is necessary to determine the effectiveness of the learned models against older adults and people with sleep disorders. The findings here should also not be considered a conclusive evaluation of which transfer learning algorithms or measures necessarily work best in all instances, as we used a single target domain with similar source domains in a supervised setting. Many of the transfer learning algorithms and transferability measures were developed for computer vision tasks, for an unsupervised/semi-supervised setting, or when using engineered instead of learned features, and so more research is necessary to determine whether the results found here are true of other tasks, other source/target combinations, other sensors or other settings. However, our findings do highlight that the source data and target data influence both the type of transfer learning approach and the measure for identifying the best approach.

## Conclusion

It was experimentally found that the most widely used transfer learning method (re-training the head layers) was the most robust approach, as it was either the best or second best in all three experimental conditions. DDC however was able to out-perform head re-training in one case, but showed considerable sensitivity to the choice of layers to which it is applied. H-score was correlated best with accuracy in cases where the assumption of a fixed feature extractor is met, but this assumption is violated for transfer learning methods which involve re-training all layers and for architectures with state-dependence. TDAS can be a strong correlate with accuracy across cases, but shows some sensitivity to the choice in the $\epsilon$ parameter. Future research directions could include training different layers with different learning rates to see how this impacts each algorithm and measure, investigating the characteristics of the data that make some algorithms more effective than others, and testing in semi- or fully unsupervised settings.

## Materials and methods

### Datasets

CiCC contains 994 healthy subjects or patients experiencing spontaneous arousals, respiratory effort related arousals, bruxism, hypoventilation, hypopneas, apneas, vocalizations, snores, periodic leg movements, Cheyne–Stokes breathing or partial airway obstructions. SHHS contains 6441 healthy subjects or patients with atherosclerosis, airway obstructive diseases or other cardiovascular problem. ISRUC contains 126 healthy subjects and patients with various disorders including REM sleep behavior disorder, obstructive sleep apnea, snoring, periodic limb movement, epilepsy, depression, Parkinson’s or insomnia. MrOS contains 2900 healthy subjects and patients with sleep disordered breathing or nocturnal hypoxemia. WSC contains over 1100 healthy subjects and patients with sleep disordered breathing. The MASS dataset contains 200 subjects with apnea/hypopnea indices of up to 20 but were otherwise healthy except for 15 with mild cognitive impairment and 7 with restless leg syndrome. In order to avoid biasing the model towards subjects who had done more recordings, only the first recording from every subject was used when training on datasets in which some subjects had multiple recordings. In all datasets, a single EEG channel was used. Either the C3-M2 or C3-A2 EEG channel was used (depending on which was available) in the source datasets in order to keep the signal content as similar as possible to the channels available in the target dataset, which were across the forehead. The target dataset contains 24 subjects with 1–6 recordings each (75 recordings total) using an X4 Sleep Profiler (Advanced Brain Monitoring, Carlsbad, CA). The X4 consists of a headband and several sensors across the forehead. A single channel (AF7-Fpz) was used. Ground truth sleep stage labels were manually determined by a human sleep staging technician. Subjects were volunteers recruited from Georgia Tech’s graduate student body and from Emory’s Biomedical Informatics department. As with the source dataset, no more than two recordings per subject were included in the training set in order to avoid biasing the model towards subjects who volunteered for more recordings. However, no data were excluded during evaluation.

### Training and testing procedure

To evaluate the effectiveness of each transfer learning algorithm, six base models using each of the two architectures (12 models total) were pre-trained on one of the six source datasets before being re-trained on the target dataset using one of the transfer learning algorithms described above. The pre-training procedure for the open-source model was done using the same methods and parameters described in [43]. The bespoke model was pre-trained using ADAM with an initial learning rate of 0.001 either for 1000 epochs or until the early stopping criteria (running 30 epochs without obtaining more than a .1% improvement in accuracy on a validation set) were met, whichever came first. Dropout at a rate of .5 was applied to the fully connected layer and all layers used an L2 regularization weight $10^{-6.9}$ (values found using Bayesian hyperparameter tuning). Transfer learning hyperparameters for both architectures were the same as the hyperparameters used in pre-training the bespoke model. For source datasets where some subjects had multiple recordings, only the first recording from each subject was used in order to avoid biasing the model towards the subjects with multiple recordings. Some of the transfer learning algorithms required training on source and target simultaneously; in these cases, a subset of source recordings equal to the number of target recordings were selected so as to maintain a balanced number of source and target samples. The subset of source recordings was selected by choosing every nth recording in the order in which they were numbered to ensure the subset was representative of the full dataset. The average accuracy and Cohen’s $\kappa$ were obtained using leave-one-subject-out cross-validation. The number of times a particular transfer learning algorithm outperformed every other learning algorithm for a particular validation subject was also tallied. A paired t-test was used to determine whether each transfer learning algorithm significantly outperformed the baseline method of head re-training.

Transferability measures were evaluated only on the target subjects used in training—measures were not evaluated on the subject being left out for validation. The relationship between each transferability measure and the performance of the re-trained model was evaluated using Spearman’s rank correlation between the transferability measure and the accuracy of the pre-trained model on the validation set. A high correlation with accuracy suggests the transferability measure is a reasonable indicator of how well a particular model will perform on the dataset. The reason Spearman’s correlation was used instead of Pearson’s correlation is because the transferability measures do not necessarily increase linearly with the accuracy of the trained model. The overall correlation between accuracy and each transferability measure is found along with the correlation for each individual transfer learning algorithm.

### Inclusion of women and minorities

In compliance with Sex and Gender Equity in Research (SAGER) guidelines, we report the demographic makeup of all datasets. MrOS is the only dataset which is entirely male. ISRUC is 40% female (47 female patients total), WSC is 46% female (515 patients total), CiCC is 34% female (227 female patients total), and SHHS is 52% female (3039 patients total). The SHHS is 86% White, 9% Black, and 7% Other. MrOS is 93% White, 4% Black, 3% Asian, 0.1% Native American, Native Hawaiian or Native Pacific Islander, 1% Multiracial and 2% Unknown. The WSC is 94% White, 2% Black, 1% Asian, 1% Hispanic and 1% Native American. CiCC and ISRUC did not report the racial distribution, but are obtained from hospital PSG records without regard for race, and are thus expected to reflect the racial distribution of patients referred to sleep labs.

### Base models

Two base models were used—one bespoke model of our own design (Fig 1) and an open-source architecture, DeepSleepNet [43] (Fig 2). DeepSleepNet was selected due to its state-of-the-art performance on sleep staging tasks, for its frequent use as a basis of comparison by other papers [22, 44–46], and for having a much larger, and differing architecture from our bespoke model. Furthermore, DeepSleepNet uses a combination of both convolutional and recurrent layers. The convolutional layers extract features from one epoch while the recurrent layers take temporal information into account (i.e., the score of one epoch is used for determining the score of the subsequent epoch). Most state-of-the-art architectures employ a similar paradigm of combining feature-extracting convolutional layers with recurrent layers or transformer mechanisms [45–47, 54–60], and so DeepSleepNet is representative of state-of-the-art methods. For the bespoke model, 4 convolutional layers were used, with each convolutional layer being followed by a ReLU, Max Pooling and Batch Normalization layer. The input was the short-time Fourier transform of the EEG. The head of the model was a single dense layer. Dropout was used on the dense layer. Training was done using the Adam optimizer [61]. The dropout fraction, L2 regularization and number of filters in each layer were found using Bayesian hyperparamter tuning on the SHHS dataset. Training and testing on the clinical datasets showed the base model to be capable of performance on par with that of human technicians (Table 13).

DeepSleepNet consists of two branches containing a series of convolutional, batch normalization, ReLU, max pooling and dropout layers which then merge before being fed into two more separate branches, one containing a fully connected layer and the other containing two consecutive bi-directional LSTM layers which are each followed by a dropout layer. The two branches are then merged and fed into a softmax layer. The input is raw EEG. DeepSleepNet uses two phases of training. In the first phase, the convolutional layers without the LSTM layers are trained for 100 epochs on a version of the dataset which was class-balanced by randomly duplicating samples from minority samples. In the second phase, the LSTM and final softmax layers were added and the model trained again for 200 more epochs on the original imbalanced dataset in batches of 25 consecutive epochs. In the original paper, the performance of DeepSleepNet is evaluated on the test set every epoch and the model weights from the epoch which achieved the highest accuracy on the test set are used to test the model again on the same subject, with the only difference being that the model states are re-set in between each 25-epoch batch during training. DeepSleepNet was trained on each source dataset using the most of the same code and parameters used in the original DeepSleepNet paper, but transfer learning was performed using the most of the same code and parameters as our bespoke model. The subjects used for early stopping during re-training are separate from the subjects used for testing. During pre-training, 1% of subjects are separated from the rest of the source subjects and used to decide at what training epoch to load the highest-performing weights from.

### Re-training larger numbers of layers

To test how the choice of layers to re-train effects the performance of each algorithm and transferability measure, each algorithm was applied to the convolutional layer closest to the output in addition to just the dense layer. For the head re-training algorithm, the dense layer, the nearby convolutional layer and each of their respective batch normalization layers were re-trained while the other layers were frozen. For CORAL, Per-Class CORAL, and SA, the same layers were frozen and re-trained as with Head Re-train, but an additional domain adaptation was applied to the output of the frozen layers. For DDC, all layers were re-trained, but the MMD loss was applied to input to the max pooling layer second closest to the output layer.

Note that only one set of layers was tested for DeepSleepNet. Because DeepSleepNet makes use of multiple branches which split and re-join, there is no location within the architecture to apply the domain adaptation/loss function which can be compared to a similar location within the more linear bespoke CNN except for the output layer.

## Data Availability

The code used for analysis is available in our public github repository. The wearable device datasets used during the current study will be made publicly available once consent to publish the data is obtained from all subjects. The clinical PSG datasets ISRUC [62] and CiCC [63] are publicly available from their respective websites. All other hospital PSG datasets are available from their respective owners but restrictions apply to the availability of some of these datasets, which were used under license for the current study, and so are not publicly available. Data are, however, available from the authors upon reasonable request and with permission of the owners of each dataset.

## Appendix

See Table 13.

**Table 13:** Fivefold cross-validation of neural network trained and tested on source datasets without transfer learning

Dataset	Accuracy (%)	Cohen’s $\kappa$
SHHS	80.8	0.725
WSC	83.6	0.731
CiCC	72.7	0.634
MrOS	85.5	0.774
ISRUC	73.7	0.66
MASS	79.8	0.713

## Abbreviations

EEG: Electroencephalography
ECG: Electrocardiography
REM: Rapid eye movement
CORAL: Correlation alignment
DDC: Deep domain confusion
SA: Subspace alignment
SHHS: Sleep Heart Health Study
CiCC: Computing in Cardiology Challenge 2018
ISRUC: Institute of Systems and Robotics, University of Coimbra
MASS: Montreal Archive of Sleep Studies
MrOS: Osteoporotic Fractures in Men Study
WSC: Wisconsin Sleep Cohort
SAGER: Sex and Gender Equity in Research
ReLU: Rectified linear unit
MMD: Maximum mean discrepancy
PCA: Principal component analysis
LEEP: Log expected empirical prediction
TDAS: Target density around source
